# Find the Word! — But Where?: Maturana’s ‘Coordination’ and Sartre’s ‘Reflection’ around Naming

**DOI:** 10.3389/fpsyg.2015.01814

**Published:** 2015-11-24

**Authors:** Seiichi Imoto

**Affiliations:** Independent ResearcherSapporo, Japan

**Keywords:** J.-P. Sartre, H. R. Maturana, languaging, naming, coordination, pure reflection, impure reflection, synthesis of identification

## Abstract

‘Behavioral coordination’ theory of language of Maturana (1928–) does not give a clear explanation for the questions of how naming takes place and where a word adequate for our experience comes from. This flaw may be alleviated by Sartre (1905–1980)s ‘reflection’ theory. According to Sartre’s theory, we can make two types of sentences from the same data: for example, “I am conscious of this chair” and “There is consciousness of this chair.” The difference between the two sentences is the existence of ‘I’ in the first or its lack in the second. Where did ‘I’ come from or how was it removed? There must be a field in which ‘I’ is brought forth, and it may also be a field where naming can take place. This essay concerns a naming process with special reference to Sartre’s philosophy. At first, Maturana’s biology and his linguistic theory are explained, and Sartre’s fundamental ontology and in relation to this, his theory of reflection (two types of reflection) are introduced. Next, Sartre’s notions of language (words and naming) are explained. Then, after operational correspondences between Maturana’s ‘coordination’ and Sartre’s ‘reflection’ are examined, our primary questions are answered. Finally, constraints burdened on our cognition with language and the possibility of liberation from them are discussed. Main arguments: (1) Maturana’s ‘coordination’ and Sartre’s ‘reflection’ are operationally equivalent concepts; (2) Sartre can complement Maturana’s languaging theory of naming by providing both the domain for naming (the domain for the synthesis of identification, or for universalizing synthesis) and a mediator of naming (the cogito, namely the consciousness, of a languaging person).

## Introduction: Proposition Of The Problem

Maturana and Sartre — most readers may be surprised or perplexed with this juxtaposition. At first glance, commonsensically, they don’t seem to have anything to share. However, actually they have many commonalities: religiously they are both atheists, and politically both are anarchists seeking for love or solidarity among people. Moreover, in their work the two are fundamentally phenomenological ontologists, and they share many common problematics: the nature of perception, illusion, imagination, and emotion; phenomenological ontology of consciousness, self (the ego) and self-consciousness, and so on.

I have been studying Maturana’s work for years (cf. “The logic of Maturana’s biology,” 2011; “What is H. Maturana’s ‘Languaging’?,” 2013, inter alia). It is when I began to examine Maturana’s notions of ‘emotion’ and ‘emotioning’ that I encountered Sartre’s work, and consequently I found many commonalities between them as noted above.

Maturana’s ‘biology’ is not an ordinary biology as a natural science. It should be called meta-biology or second-order biology. Usually its description is abstract, formal, and lacks concrete examples. In the meanwhile, I found Sartre’s description could give us vivid and concrete examples to flesh out some of Maturana’s notions. For example, the notion of ‘structural coupling’: the dynamics of congruent structural changes that take place spontaneously between systems in recurrent (in fact recursive) interactions (Maturana, 2002, p. 17). We can have a vivid experience of the structural coupling by reading Sartre’s following sentences in his *Being and Nothingness* (Sartre, 2003, pp. 605–606).

*The skier makes it [=the snow] produce* what it can produce; the homogeneous, solid matter releases for him a solidity and homogeneity only through the act of the sportsman, but this solidity and this homogeneity dwell as properties enclosed in the matter. This synthesis of self and not-self which the sportsman’s action here realizes is expressed, as in the case of speculative knowledge and the work of art, by the affirmation of the right of the skier over the snow. It is *my* field of snow; I have traversed it a 100 times, a 100 times I have through my speed effected the birth of this force of condensation and support; it is *mine*. (italics in original)

“It is my field of snow and the snow is mine!” I think this is a very beautiful expression of Maturana’s notion of structural coupling. Through that, it can be said that Sartre complemented Maturana very effectively. This can be the case with Maturana’s other problematics. As one of them, I have adopted a linguistic problem, that is, the problem of naming.

### Maturana’s Behavioral Coordination Theory of Language

In his talk with Poerksen, Maturana spoke about his behavioral coordination theory of language (Maturana and Poerksen, 2004, p. 91).

I claim that whenever we encounter a recursive coordination of behavior, that is, a flow in coordination of coordinations of behavior, we see that something new arises, namely, language. As language arises, objects arise, e.g., the taxis. What is a taxi? What I say is that carrying and driving around passengers as a configuration of behavior coordinated by the second coordination of behavior (first recursion), becomes that configuration of behavior that in a third coordination of behavior (second recursion) appears “named” taxi. This means that objects arise as coordinations of coordinations of behavior that obscure the behaviors that they coordinate (as taxi obscures carrying).

Maturana’s ‘language’ means ‘coordinations of coordinations of behavior’ in the consensual domain, therefore, it is also properly called ‘languaging.’ In languaging, the first coordination of behavior brings forth ‘carrying and driving around passengers,’ the second ‘an object (as a would-be taxi),’ and the third ‘an object named taxi.’ Here, I have two questions: (1) Where did the name ‘taxi’ come from? Was it created from scratch through those coordinations, or was it found somewhere and borrowed to apply to the object?; (2) Who coordinated and named the object as ‘taxi’? Since languaging is not a natural phenomenon, the phenomenon of naming will not occur spontaneously or automatically; some subject (agent) has to be involved in that process. To my knowledge, Maturana’s description of languaging has never seriously concerned such problems in naming.

### Sartre’s Two Types of Sentences derived from the Same Data

Let’s assume there is a chair in front of Sartre. He makes two sentences from that same situation (Sartre, 2011, p. 16).

(1) I am conscious of this chair.
(2) There is consciousness of this chair.

The difference between these sentences is the existence of ‘I’ in the sentence (1), or its lack in the sentence (2). What makes this difference? Where does ‘I’ come from? Or how can ‘I’ be removed?

The naming of ‘taxi’ and the appearance and disappearance of ‘I’ may be connected. There may be a field in which ‘I’ is brought forth and the naming of ‘taxi’ may also take place. Sartre’s philosophy could explain this problem and complement Maturana’s behavioral coordination theory of language.

## Maturana’ ‘Biology’ (see Imoto, 2011, 2013)

Maturana’s ‘biology’ or a second-order biology is a philosophy of structural determinism. It is embodied in a composite entity called a ‘structure-determined system’ with two non-intersecting domains – the domain of interactions and the domain of the composition of components – which can be called the core structure or core logic of his biology. From the perspective of the history of western thoughts, Aristotle and Schopenhauer can be regarded as good candidates as precursors of Maturana’s work, and his work can be characterized as an advanced form of Aristotle’s hylomorphism (Aristotle, 1984), depicted on the horizon of Schopenhauer’s world of ‘Vorstellung’ (representation or bringing-forth; Schopenhauer, 1966, 1997).

By putting two organisms and their niche (which are all structure-determined systems) into a historical process, Maturana develops his core logic of the structure-determined system, letting biological phenomena (for example, language) arise from them in the domain of interactions of the observer. All the findings observed by the observer, hence, exist under the consciousness of the observer observing, in other words, on the same epistemological horizon as that of Schopenhauer’s world of Vorstellung. Ontologically Maturana’s universe is composed of the observer’s consciousness and his all findings observed under his consciousness. This ontological structure is fundamentally the same as that of Sartre’s which is described later.

Maturana developed a quite unique theory of language. He wrote (Maturana, 1995, p. 155): when an observer sees two organisms in a flow of recurrent interaction that he or she can describe as consensual coordinations of consensual coordinations of behavior, those two organisms operate in language. Thus, according to him, our language occurs in *languaging* as a flow of living together in coordinations of coordinations of consensual doings (actions or behaviors) in the domain of interactions (or in the consensual social domain).

### Languaging as Organization of Language

What is the nature of ‘languaging,’ or what kind of status does it take in Maturana’s system of language? Maturana writes about ‘natural language’ as follows (Maturana, 1980, pp. 30–31).

The understanding of the evolutionary origin of natural languages requires the recognition in them of *a basic biological function which, properly selected, could originate them*. So far this understanding has been impossible because language has been considered as a denotative symbolic system for the transmission of information. In fact, if such were the biological function of language, its evolutionary origin would demand the pre-existence of the function of denotation as necessary to develop the symbolic system for the transmission of information, but this function is the very one whose evolutionary origin should be explained. Conversely, if it is recognized that language is connotative and not denotative and that its function is to orient the orientee within his cognitive domain, and not to point to independent entities, it becomes apparent that learned orienting interactions embody a function of non-linguistic origin that, under a selective pressure for recursive application, can originate through evolution *the system of cooperative consensual interactions between organisms that is natural language*. (italics by s.i.)

For Maturana, ‘natural language’ is ‘the system of cooperative consensual interactions between organisms,’ in other words, it is the ‘languaging’ as ‘coordinations of coordinations of consensual doings,’ and thus, without languaging there will be neither languages nor even symbolic systems. An explanation is, for him, the proposition of a generative mechanism or process which, if allowed to operate, gives rise, as a result of its operation, to the phenomenon or experience to be explained. So, he proposed the autopoietic organization as the generative mechanism that brings forth living phenomena of living systems in their niche, and the closed circular neuronal organization of the operation of the nervous system as the generative mechanism that brings forth and modulates sensory-motor (or effector) correlations, hence behaviors, in the organism. Thus, to find a generative mechanism in a system in question is to find an organization as an identity or essence of the system. Languaging, then, as a basic biological function in the domain of interactions, is the generative mechanism or the organization as essence of the linguistic system in general and of the human language system in particular.

### Maturana’s Linguistic Theory

Maturana summarized his linguistic theory as follows (Maturana, 2008, pp. 19–20).

(1) If we attend to what we do in language, we will realize that language occurs as a flow of living together in coordinations of coordinations of consensual doings. That is, we will realize that language occurs as languaging, in the flow of our living together in recursive consensual coordinations of doings. Language has the concreteness of the doings in the domain of doings [=the domain of interactions] in which we coordinate our doings.
(2) Objects, entities, notions, ideas, concepts, etc., arise as coordinations of coordinations of doings, and do not exist otherwise. Meanings of words, sentences, signs, and symbols are not in them, but in the flow of coordinations of doings that they coordinate. And a word can have as many different meanings as there are different flows of recursive coordinations of doings in which the word participates.
(3) When a child learns to name an object he or she does not learn to name a preexisting entity, but learns a flow of recursive coordinations of doings with languaging persons with which he or she may be living. So a baby that learns the [name of] ball, learns balling [ball-ing], and when he or she learns the [name of] doll, learns dolling [doll-ing]. Thus, the baby learns them as manners of living together with other human beings in consensual coordinations of doings.

The item (3) above provokes in me the same questions as noted in the case of ‘taxi’: (a) where do such words (or names), ‘ball’ and ‘doll,’ come from in his linguistic theory? Why can Maturana name those doings, balling and dolling, as such? Can the consensual coordinations of doings create such words as ‘ball’ and ‘doll’? Or, are there already such words as the given in the consensual domain of interactions for children to be able to learn and use them?; (b) If a word (ball) and its meaning (ball-ing) are given there, do they combine automatically or isn’t some agent required to combine them? Maturana’s account of naming as the (3) above seems insufficient to me. If the languaging is truly the generative mechanism or the organization of the natural language system, it should give a clearer account of the emergence of words or of naming.

## Sartre’s Philosophy

Now I refer to Sartre’s work “*The Transcendence of the Ego: A Sketch for a Phenomenological Description*” which was published in 1937. Although this is one of his earliest works, we can already find in it his fundamental philosophical principles underlying even his later major works such as “*Being and Nothingness*” (1943) and “ *Critique of Dialectical Reason, Volume One*” (1960).

### Ontology

He began “*The Transcendence of the Ego*” by writing as follows (Sartre, 2011, p. 1).

For most philosophers, the Ego is an ‘inhabitant’ of consciousness. Some of them state that it is formally present at the heart of ‘Erlebnisse [lived experiences],’ as an empty principle of unification. Others – psychologists, for the most part – claim they can discover its material presence, as a center of desires and acts, in every moment of our psychical life. I should like to show here that the Ego is neither formally nor materially *in* consciousness: it is outside, *in the world*; it is a being in the world, like the Ego of another. (italics in original)

He exiled the Ego from the consciousness to the world outside it (in Maturana’s terms, it can be rephrased like this: Sartre exiled the *Self* from the domain of the composition of components to the domain of interactions). Thus, the consciousness was cleaned and purified. He wrote in the first part of the Conclusion of that book as follows (Sartre, 2011, p. 43).

(1) The transcendental field [=the consciousness, by s.i.], purified of all egological structure, recovers its former limpidity. In one sense, it is a *nothing*, since all physical, psycho-physical and psychical objects, all truths, and all values are outside it, since the *me* has, for its part, ceased to be part of it. But this nothing is *everything* because it is the *consciousness* of all these objects. (…) But, in addition, we have to note that, from this point of view, my feelings and my states [of mind], my Ego itself, cease to be my exclusive property. (italics in original)

For Sartre, the two domains, that is, the consciousness (*my* consciousness) and the world outside it, constitute all the universe of human existence. What is important here is that the consciousness is the consciousness of the world. The world contains everything except my consciousness: not only all the physical and psycho-physical but also all the truths (e.g., mathematical truths) and values, and in addition, the Ego (the I and the me) and its related feelings and states, and all other psychical objects are all existents in the world, not in my consciousness. As a result, he refuted the solipsism.

The Ego appears to reflection as a transcendent object in the world (Sartre, 2011, p. 28). The Ego, the unity of transcendent unities such as mental states, qualities and actions, is itself a transcendent, and appears only in the world of reflection (Sartre, 2011, p. 21).

Consciousness is defined by intentionality, through which, in Sartre’s terms, it transcends itself to the intentional object; the object is transcendent to the consciousness that grasp it, and it is within the object that its unity is found (Sartre, 2011, p. 6).

Thus, he reaches the last part of his Conclusion (Sartre, 2011, p. 51).

(3) It is sufficient for the *me* to be contemporary with the World and for the subject-object duality, which is purely logical, to disappear definitively from philosophical preoccupations. The World did not create the *me*, and the *me* did not create the World, they are two objects for the absolute, impersonal [which means ‘without the Ego,’ by s.i.] consciousness, and it is through that consciousness that they are linked back together. (italics in original)

He could exile, in addition to the solipsism, the subject-object duality as only logical. Now here are the absolute, impersonal consciousness in one side, and the Ego and the world in the other side: in reflection, the consciousness brings forth the Ego into the outside world, both of which are supported by the impersonal consciousness. Simply put, as noted above, Sartre’s ontology is composed of the two fields: *my* consciousness and the world outside my consciousness, in other words, the world and my consciousness of it.

The ontological situation for Maturana is generally the same as that for Sartre. Everything arises in languaging coordinations: in addition to objects, ideas, concepts, etc., the observer (the self), consciousness, self-consciousness are brought forth in the consensual domain of interactions (Maturana, 1992, 1995). And, since it is the observer that sees all these entities, the world appears as the world of Vorstellung of Schopenhauer (1966, 1997) to the observer’s consciousness. This ontological structure for Maturana fundamentally the same as that of Sartre’s noted above.

### Two Types of Reflection

Sartre distinguishes two types of reflection, impure and pure. When these two reflections apprehend the same, certain data, impure reflection affirms more than it knows (a process called ‘infinitization’ or ‘universalization’), but pure reflection stays with the given (no infinitization; Sartre, 2011, p. 23).

In *Being and Nothingness* Sartre referred to the two types of reflection as follows (Sartre, 2003, p. 182).

Here, we must distinguish between pure reflection and impure or constituent reflection, for it is impure reflection which constitutes the succession of psychic facts or *psyche*. What is given first in daily life is impure or constituent reflection although this includes pure reflection as its original structure. But pure reflection can be attained only as the result of a modification which it [impure reflection, by s.i.] effects on itself and which is in the form of a katharsis. (italics in original)

So, reflection is usually carried out in the following sequence: (1) pure reflection, which is mostly hidden, (2) impure reflection which includes the (1) as its original structure, and (3) pure, or rather purifying, reflection in the form of a katharsis (actually in the form of phenomenological reduction or bracketing off the Ego).

Let’s see a concrete example to clearly understand the reflection process, especially of the (1) and (2) above. Sartre shows us a reflective experience of hatred as follows (Sartre, 2011, pp. 22–23). You will also see what he meant by ‘infinitization.’

Let us consider a reflective experience of hatred. I see Peter, I feel a kind of profound upheaval of revulsion and anger on seeing him (I am already on the reflective level); **this upheaval is consciousness.** I cannot be in error when I say: I feel at this moment a violent revulsion toward Peter. But is this experience of revulsion hatred? Obviously not. It is in any case not given as such. After all, I have hated Peter for a long time and I think I always will hate him. So an instantaneous consciousness of revulsion cannot be my hatred. (…) I would say: ‘I feel revulsion for Peter *at this moment*,’ and in this way I will not implicate the future. But precisely because of this refusal to implicate the future, I would cease to hate.

But my hatred appears to me at the same time as my experience of revulsion. But it appears *through* this experience. It is given precisely as not being limited to this experience. It is given, *in* and *by* each movement of disgust, revulsion and anger, but at the same time it is *not* any of them, it goes beyond each of them as it affirms its permanence. (…) This is enough, it seems to me, for one to be able to affirm that **hatred is not *a form* of consciousness**. It extends beyond the instantaneous moment of consciousness (…). **Hatred is thus a transcendent object**. Each *Erlebnis* reveals it in its entirety but at the same time is merely a profile of it, a projection (an *Abschattung*). **Hatred is a letter of credit for an infinity of angry or revulsed consciousnesses, in the past and future**. It is the transcendent unity of that infinity of consciousnesses. So, to say ‘I hate’ or ‘I love’ on the occasion of a singular consciousness of attraction or revulsion is to perform a veritable infinitization, somewhat analogous to the one we carry out when we perceive *an* inkwell or *the blue* of the blotter. (italics in original; boldface by s.i.)

Revulsion is a revulsed consciousness; hatred is in the field outside consciousness as a psychical state, as a transcendent object. Revulsion is in instantaneousness of time; hatred in infinitization or in infinity, independent of time. Impure reflection (hatred), thus, includes pure reflection (revulsion) as its original structure. The step (3) of reflection, purifying reflection, is to take the impure reflection back into its original instantaneous character, in other words, into the level of original unreflected consciousness.

## Sartre’s View Of Language

### Words

Sartre regards words and language in general as a kind of ‘pratico-inertia’ (matter in which human past praxis is embodied as inertia). They are in the practico-inert field of the field outside consciousness, surrounding and conditioning us (Sartre, 2004, p. 324).

Every word is external to everyone, it lives outside as a public institution (Sartre, 2004, p. 98). But language is the product of human history (Sartre, 2004, p. 99), so its materiality as an inert totality is a constantly developing organic totalization (Sartre, 2004, p. 98). So, we can say that the practico-inert field which contains words and language, is, in Maturana’s terms, in the consensual domain of interactions, in which all aspects of language (lexis, syntax, semantics, and pragmatics) have been brought forth and developed as an organic totalization.

### Naming

#### Three Requisites for the Commencement of Language

Here, I would like to refer to “There and Back” (Aller et Retour). This is Sartre’s essay on language presented in the form of a critique on Parain (1897–1971)’s *Recherches sur la nature et les functions du language* (Paris: Gallimar, 1942). In this essay, Sartre argues three requisites for language: the *cogito*, the universalizing synthesis, and the Other (Sartre, 2010a, p. 368).

Against Parain, then, we must maintain the priority of the *cogito*, of universalizing syntheses, and of immediate experience of the Other. In this way, we restore language to its true place. (italics in original)

What is the *cogito* in this case? It is the consciousness, the being-for-itself, which brings forth the ‘I’ in the field outside it. Sartre explains (Sartre, 2010a, pp. 363–365).

[T]he word, the sole word in question is there *before* **me** as *that which is understood*. (…) [T]he consciousness of understanding is the law of being of understanding. I shall call this the silence of consciousness. (…) [T]hat silence that I am, by which, however, there is a language and there is a world. (italics in original; boldface by s.i.)

Through **me** (i.e., my consciousness, the cogito), there are the world and language in it. This is the same structure as was noted above, i.e., there are the Ego and the world, which are linked back together by the consciousness, the cogito. In Maturana’s terms, this consciousness (the cogito) can be that of a languaging person as far as languaging is concerned. Thus, his or her cogito turns out to be a mediator through which the world and language are linked back together.

Then, Sartre asked: which is first, the Other or language? (Sartre, 2010a, p. 365). He replied that language is being-for-others: the Other – any other – comes in between me [the cogito, my consciousness] and everything I am on Earth – happy, unhappy, handsome, ugly, mean or magnanimous, for the Other must play a part before I can be any of those things (Sartre, 2010a, p. 367). He further noted (Sartre, 2010a, p. 366):

But if I exist originally only by and for the Other, if, as soon as I appear, I am thrown before the Other’s gaze; and if the Other is a thing as certain to me as I am myself, then I am language, for language is merely existence in the presence of someone else. (…) In a word, for there to be a problem of language, the Other must first be given.

For Sartre’s linguistic view, the existence of other persons is one of the requisites for language. This is also the case with Maturana, because languaging requires at least two persons.

#### Domain of Naming

The last requisite to note for language is universalizing synthesis. This is the very phase of naming for which Maturana has never given a clear explanation. The domain of universalizing syntheses is just the domain of naming. Sartre asks (Sartre, 2010a, p. 308), “How is one to insert into language an experience that was made without it?” In order to do that, we need a synthetic domain of identification between language and experiences (objects, concepts, or things). Sartre explains (Sartre, 2010a, pp. 361–363).

[E]ven if the word existed in the heart of God, I would have to produce it by the operation termed ‘**synthesis of identification.**’ And I now understand that the word wasn’t privileged, since I have also to make the table and the tree and the cockchafer bug exist as permanent syntheses of relatively stable properties. It isn’t by naming them that I confer objectivity on them, but I cannot name them unless I have already constituted them as independent units or, in other words, unless I objectify both the thing and the word in a single synthetic act that names it. (…) [I]t is I [the cogito], whether listening or speaking, who constitute the word as one of the elements of my experience. (…) [U]ltimately, if I constitute my experience and words within that experience, it isn’t at the level of language, but at the level of the synthesis of identification that **the universal** appears. When I say, ‘I’m hungry,’ then clearly the word universalizes; but in order to universalize, it must first be the case that *I* [the cogito] individualize it, that is to say, that I extract the word ‘hungry’ from the disordered confusion of my current impressions. (italics in original; boldface by s.i.)

For naming to occur, it is necessary that there is a domain for universalizing synthesis, or a domain for synthesis of identification where the universal appears, in which there must be the thing (independent unit, object, or concept), the word fitting it, and the cogito, ‘I,’ that extract the word and the thing for a synthetic act of identification. The thing and the word are all inhabitants in the field outside consciousness, and therefore, the domain for universalizing synthesis or simply the domain of naming is also in that field outside consciousness, which are all supported in impure reflection through the consciousness (the cogito).

We need the cogito, the consciousness of a languaging person, as a mediator between language and experiences (objects, concepts, or things) to insert them into language, or in other words, to connect experiences with words, or simply put, to name experiences using words. Even if there are only experiences and words, they cannot automatically give rise to the phenomenon of naming. Naming needs a mediator, namely, the cogito of a languaging person.

Such are what Maturana lacks in his languaging theory of naming. In his own account of naming, he has referred neither to the involvement of the cogito in the naming process, nor to the presence of the domain of the synthesis of identification or the domain of universalizing synthesis. Sartre can complement Maturana’s languaging theory of naming by providing both the domain for the synthesis of identification, namely the domain of naming, and the cogito (the consciousness) of a languaging person as a mediator of naming.

Words are situated between objects and the cogito of a languaging person who names them. Hence, Sartre argues (Sartre, 2010a, p. 372), “language can lie, deceive, distort, and make unwarranted generalizations: the questions it raises are technical, political, esthetic, and moral.” Thus, naming can be dangerously a multi-faced project.

## Operational Correspondence Between Maturana’s ‘Coordination’ And Sartre’s ‘Reflection’

### Literal Explanation

Now I would like to show the equivalence as operational concepts between Maturana’s ‘coordination’ and Sartre’s ‘reflection.’ At first, it can be explained literally, which takes a chain of equivalences as follows: coordination of behavior (action, doing) (Maturana) = coordination of distinction (Maturana) = positing (Sartre) = reflection (Sartre).

Maturana (1992, p. 93) wrote:

I speak of coordinations of actions or coordinations of distinctions, depending whether I want to emphasize what takes place in the interaction in relation to the participants (coordination of actions), or what takes place in the interaction in relation to an environment (coordination of distinctions).

In a strict sense, the participants are also the environment, and the environment the participants. Hence, ‘coordination of actions’ and ‘coordination of distinctions’ are fundamentally equivalent.

Sartre (2010b, p. 187) wrote:

[E]very existent, as soon as it is posited, is consequently surpassed. But still it must be surpassed *toward something*. (italics in original)

In order to posit something, it must be distinguished, namely, in Sartre’s terms, it must be surpassed toward the something, or it transcends itself to the intentional object, and in order to be distinguished, it must be posited; so, positing is distinction, and vice versa.

In Sartre’s writing, ‘positing,’ ‘thesis,’ and ‘reflection’ have equivalent usage (meaning) like such expressions as ‘positional (or thetic, reflective) self-consciousness’ (Cox, 2008, p. 189). Hence, ‘positing’ is equivalent to ‘reflection.’

Therefore, entirely viewing, Maturana’s ‘coordination’ turns out equivalent to Sartre’s ‘reflection.’

### Substantial Explanation

In addition to the literal explanation above, another type of explanation which can be called a substantial explanation is possible to show the equivalence of Maturana’s ‘coordination’ and Sartre’s ‘reflection.’

Sartre said, as quoted above, that every existent, as soon as it is posited, is **consequently** surpassed. But still it must be surpassed *toward something* (italics in original; boldface by s.i.). Maturana describes this process as follows (Maturana, 1992, p. 92).

[B]ecause an observer can describe such a domain of recurrent interactions in semantic terms, by **referring** the different coordinations of **actions (or distinctions)** involved **to** the different **consequences** that they have in the domain in which they are distinguished, I also call a consensual domain of interactions a linguistic domain. [boldface by s.i.]

Simply put, this quote can be that the coordination of actions (or distinctions) is referred to its consequence. For example, ‘balling’ (as a meaning) as the coordination of actions (or distinctions) is referred to ‘ball’ (as a word) as its consequence. In Sartre’s terms, as soon as a meaning (as an existent) is posited, it is consequently surpassed toward a word (as another existent) that will take that meaning. Again in Maturana’s terms, this process can be expressed like this: words obscure their meanings (Maturana, 1995, pp. 154–155). Generally speaking, the product (consequence) obscures its process (actions or distinctions) through which it is brought forth.

Now we could recognize that Maturana’s ‘coordination’ and Sartre’s ‘reflection’ are operationally equivalent concepts.

### Actual Operational Correspondences

(1) Maturana’s first coordination and Sartre’s pure reflection: as we saw above, Maturana’s first coordination of behavior results in a configuration of behavior such as ‘carrying.’ This can be compared to Sartre’s pure reflection which impure reflection includes as its original structure or to the level of unreflected consciousness, that is, the field of Erlebnis, lived experience.
(2) Maturana’s second coordination (first recursion) and Sartre’s impure reflection: Maturana’s second coordination (first recursion) yields objects unnamed yet, e.g., an object of a would-be taxi. This can be compared to Sartre’s impure reflection which brings forth the field of objects including ‘all physical, psycho-physical and psychical objects, all truths, and all values’ and words as practico-inertia.
(3) Maturana’s third coordination (second recursion) and Sartre’s universalizing synthesis: the third coordination (second recursion) of Maturana’s produces named objects: an object of a would-be taxi becomes a taxi so named. So, this stage of Maturana is for naming. Sartre’s universalizing synthesis is also for naming, and it is at this stage that Sartre complements Maturana’s behavioral coordination theory of language.
(4) Maturana’s further coordinations (recursions) and Sartre’s purifying reflection: Maturana’s coordination process increases up to the seventh coordination (sixth recursion), finally yielding ‘freedom,’ without returning back to the lower stages (Maturana et al., 1995). Thus, his coordination process does not take into account any purifying process as Sartre’s purifying reflection which will lead to a deep comprehension of a concept (word) in question.

## Revisiting The Primary Questions

### Maturana’s Case

In the first section of this paper, I asked: (1) Where did the name ‘taxi’ come from? (2) Who coordinated and named the object as ‘taxi’?

The answer to (1) should be: it was found somewhere and borrowed to apply to the object. The name ‘taxi’ was not newly invented but found, and the ‘somewhere’ should be a cultural environment in the consensual domain of interactions where there had been a name ‘taxi’ as a consensual element, namely as a practio-inertia.

The answer to (2): it must be ‘we’ who did it, because as Maturana said in the quote: whenever *we* encounter a recursive coordination of behavior (…). This ‘we,’ however, is the ‘we’ in general, then, it turns out to be the cogito of the observer, or in a more strict sense, the cogito of Maturana himself.

In the example of ‘ball’-balling or ‘doll’-dolling noted above, there are a child (or a baby) and languaging persons with whom he or she may be living. Those latter persons are most probably his or her parents. Whoever they are, it is sure they already know the words, ‘ball’ and ‘doll.’ They use (pronounce) these words when they play the ball-ing or doll-ing with their child (or baby), thus, the child (or the baby) learns the name ‘ball’ or ‘doll’ with its meaning as the doing, ball-ing or doll-ing. The words already exist as the given together with his or her parents. In doing the play of ball-ing or doll-ing, it is the cogito of the child (or the baby), not the cogito of his or her parents, that connects the name with its meaning in the domain of universalizing synthesis (Sartre) which is the domain lacking in Maturana’s languaging theory. As already noted above, in his own account of naming, Maturana has referred neither to the necessity of the cogito of a languaging person, nor to the presence of the domain of universalizing synthesis.

Sartre’s domain of universalizing synthesis can complement Maturana’s lack as the domain of naming. Sartre’s domain of universalizing synthesis is in the social domain or in the consensual domain of interactions in Maturana’s terms. In this manner, the two linguistic theories of Maturana and Sartre are well-fitted each other and there’s no category mistake between them.

Maturana proposed the languaging as the generative mechanism of natural languages such as English, Spanish, Chinese, and Japanese. These natural languages have their own lexis, syntax, semantics, and pragmatics. Therefore, Maturana’s theory, languaging, has to be able to explain their emergence, especially the emergence of lexis (words) and semantics (e.g., naming) as the most basic constituents of natural languages, which Sartre gave us as described in Section “Sartre’s View of Language.”

It seems to be a mystery that Maturana did not include a detailed explanation for naming into his behavioral coordination theory of language, because naming itself is obviously a kind of behavior, and it is an indispensable stage for linguistics.

### Sartre’s Case

The question was what made the difference between the two sentences whose cognitive contents were the same. The sentences were: (1) I am conscious of this chair. (2) There is consciousness of this chair.

The answer is clear now. The sentence (1) was made through the synthesis of ‘I’ at the site of universalizing synthesis in the level of impure reflection, and the sentence (2) was made by the purifying reflection through the phenomenological reduction of the ‘I’ of the sentence (1).

This mechanism would shed new light on the problem of variable (multi-faced) relations between cognitive contents and their linguistic expressions.

## Constraints (Alienation) By Language And Liberation From Them

I ask myself: can you create your own language as a natural language (including lexis, syntax, semantics, and pragmatics) *ex nihilo*? My answer should be ‘no’ or ‘impossible.’

I feel myself deeply situated in my mother tongue. I am tightly coupled with it. I cannot flee from it. Of course, I can make some neologies using my linguistic knowledge of prefixes, suffixes, and roots of words. But they are not pure creation; they are still rooted in my mother tongue. As Sartre (2004, 2010a) said in the quotes above, language is a product of human history, and presupposes the existence of other people; it is the being-for-others. And it exists as its totality as a set of internal objective senses; words and sentences are in their tight connections against the background of a whole of language (Sartre, 2004, p. 99). These must be the main reason why the creation of language as a natural language by individual persons is impossible.

On the other hand, there is a moment when I have a sense of alienation even from my mother tongue. It is the time when it cannot serve me to precisely express my deep experience. I feel I need more adequate words and syntax. This feeling could be the same as that of the black people born and brought up in French in France, which Sartre described as ‘negritude’ in his “Black Orpheus” (Sartre, 2008, pp. 279–280).

There is no way they will speak their negritude with precise, effective words that hit their target. There is no way they will speak their negritude *in prose*. But everyone knows that this sense of failure with regard to language considered as a means of direct expression is at the origin of all poetic experience.

The speaker’s reaction to the failure of prose is, in fact, what Bataille calls the holocaust of words. So long as we are able to believe that a pre-established harmony governs the relations between words and Being, we use words without seeing them, with a blind confidence. They are sense-organs, mouths and hands, windows opened on the world. At the first failure, this easy chatter falls away from us; we see the whole system; it is nothing but a broken, upturned machinery, its great arms still waving to *signal* in the void. We judge at a stroke **the mad enterprise of naming**. (italics in original; boldface by s.i.)

We see words, once in harmony with us, now as “the mad enterprise of naming between words and Being [entity in general].” This is the feeling that I can sense even in my mother tongue. Should I go into the world of poem as the quote connotes? No, it could not be practical for scientific and everyday use of language.

I wrote above: naming can be dangerously a multi-faced project. It “can lie, deceive, distort, and make unwarranted generalizations” (Sartre, 2010a, p. 372). This shows that in a sense, naming can be arbitrary between words and Being at the site of naming in the impure reflection. We can take advantage of that multi-faced character of naming for ‘precise and effective’ naming. Although language creation *ex nihilo* is impossible, if we pay close attention to the characters of Being and words in the site of naming, it can serve to improve our naming power.

It should be warned again that naming is brought forth in impure reflection, which can also be used as the site of forgery. That is why we need the purifying reflection to be able to affirm the sure and certain content to our consciousness. Only after understanding this, we can say, as Laing and Cooper (1971, p. 19) wrote, “language must be pressed into service even if this involves turning language against itself, exploiting its deficiencies, its vagueness, and its contradictions.”

## Conflict of Interest Statement

The author declares that the research was conducted in the absence of any commercial or financial relationships that could be construed as a potential conflict of interest.
